# The return of raptors to Scotland’s skies: Investigating the diets of reintroduced red kites and white-tailed eagles using stable isotopes

**DOI:** 10.1371/journal.pone.0315945

**Published:** 2025-01-08

**Authors:** Juliette Waterman, Stuart Black, Naomi Sykes, Andrew C. Kitchener, William F. Mills, Mark D. E. Fellowes

**Affiliations:** 1 School of Archaeology, Geography & Environmental Science, University of Reading, Whiteknights, Reading, United Kingdom; 2 Department of Archaeology and History, University of Exeter, Streatham, Exeter, United Kingdom; 3 Department of Natural Sciences, National Museums Scotland, Edinburgh, United Kingdom; 4 Department of Biological Sciences, Royal Holloway, University of London, Egham, United Kingdom; 5 School of Geosciences, University of Edinburgh, Edinburgh, United Kingdom; Senckenberg Gesellschaft fur Naturforschung, GERMANY

## Abstract

Species reintroductions are increasingly seen as important methods of biodiversity restoration. Reintroductions of red kites *Milvus milvus* and white-tailed eagles *Halieaeetus albicilla* to Britain, which were extirpated in the late 19^th^ and early 20^th^ centuries, represent major conservation successes. Here, we measured stable isotope ratios of carbon (*δ*^13^C) and nitrogen (*δ*^15^N) in feather keratin and bone collagen of museum specimens of red kites and white-tailed eagles, which were collected from across Scotland between the 1800s and 2010s. Our objectives were to investigate dietary differences between species and between the pre- and post- reintroduction periods. Among reintroduced birds, *δ*^13^C values were significantly less negative and *δ*^15^N values higher in feather keratin and bone collagen of white-tailed eagles compared to red kites, likely reflecting a greater reliance on marine resources by the former. Our stable isotope data showed a wide range, confirming the dietary diversity observed in conventional diet studies of both taxa, with white-tailed eagles, in particular, having wide dietary niches and a considerable degree of inter-individual variability. Isotopic data from pre-introduction red kites mostly fell within the range of post-reintroduction birds, suggesting they had similar diets to the pre-reintroduction birds, or the prey base for modern birds is isotopically indistinguishable from that of their historic counterparts. For white-tailed eagles, several pre-reintroduction birds were isotopically distinct from the post-reintroduction population. These differences may indicate a changing prey base, although these conclusions are complicated by shifting distributions and small population samples. Overall, our study demonstrates the utility of natural history collections in examining changes in diet, environment, and interactions with humans in reintroduced species compared with pre-extirpation indigenous populations.

## Introduction

Population numbers of red kites *Milvus milvus* and white-tailed eagles *Halieaeetus albicilla* have increased in Scotland following successful reintroductions after local extirpations [1, 2]. Prior to this, these species had close associations with human settlements, especially in medieval Britain, where foraging opportunities were rich due to waste management practices [3]. The extirpation of both species from Scotland followed centuries of persecution after the 1457 ‘Vermin Law’ passed by James II of England (James VII of Scotland), which mandated that, among other raptors, red kites and white-tailed eagles and their nests be “utterly destroyed” to protect game birds [4]. Raptor killings intensified in the 17^th^ and 18^th^ centuries, as the rise of large game-shooting estates in Scotland brought birds of prey into even greater conflict with human interests through their actual and perceived predation of gamebirds [5]. Estate records reveal the extent of raptor persecution in Scotland: 275 kites were killed at Glengarry between 1837–40 (~70 per year), and 105 were taken in the Callander Hills in 1875 alone [6]. On the Marquess of Bute’s Argyll estate, gamekeepers swore an oath to “use my best endeavours to destroy all birds of prey etc., with their nests, wherever they can be found therein. So, help me God.” [5]. Prior to reintroduction, the red kite last bred in Scotland in 1879, but its numbers were low from the 1840s [7]. By the turn of the 20^th^ century, Britain’s population of kites had dwindled to a small vulnerable number of birds, limited to remote mid-Wales [7]. The last known white-tailed eagle was reportedly shot on the isle of Skye in 1918, marking its extinction from Britain [8].

Historical and archaeological evidence for the distribution of both species contributed to the decisions to reintroduce them [8, 9]. Red kites were reintroduced to England and Scotland in 1989 with donor birds from Spain and Sweden, respectively [10]. Releases of red kites into the Chilterns in southern England and the Black Isle (highland Scotland) were successful, with the English population growing so rapidly that recently chicks were translocated from England back to Spain to reinforce the donor population [11]. The population growth rate was slower in Scotland, partly due to many instances of poisoned and shot birds [1]. Much like their medieval counterparts, red kites are now seen in many urban settlements across Britain, spreading out from the more rural landscapes to which they were originally reintroduced [12]. White-tailed eagle chicks were reintroduced to Scotland from a population in Norway to Rum between 1975 and 1985 and Wester Ross in the 1990s [8]. The highest population densities are now on Skye and Mull [13]. Food was provided at release locations to encourage the birds to visit where they could be observed [14], although released eagles habitually returned to the pens where juvenile birds awaiting their own release were kept, and additional food dumps were provided here after some ‘thefts’ [8]. The food provided included various local fish, although once released, birds often incorporated other food items such as gralloch (viscera) from shot red deer [8]. As well as official food provision by conservationists to support the reintroductions, feeding stations supplied by local landowners, where people can watch and photograph the birds, have become commonplace and popular. Reintroduced raptors can enrich local economies, such as boat tours to view white-tailed eagles feeding on Mull, which generate £5 million in local tourist spending annually [15]. The more gregarious red kite is also able to forage in urban environments; for instance, up to 1 in 20 households in Reading provide food in their gardens for red kites [16].

Raptor diets have traditionally been investigated via direct observation or analyses of crop contents, regurgitated pellets and remains from nests [2, 17]. The utility of these methods lies in their high taxonomic resolution (i.e., species-level identification of prey), but various biases can lead to the under- and over-representation of particular prey [17]. One alternative is to measure stable isotope ratios in raptor tissues, which reflect those of their prey in a predictable way [18]. Stable isotope ratios of carbon (^13^C:^12^C, expressed as *δ*^13^C) and nitrogen (^15^N:^14^N, *δ*^15^N) are the most commonly used in avian diet studies [18]. Tissue *δ*^13^C values show a limited increase with trophic level (~1 ‰), but can be used to infer the relative importance of different carbon pools (e.g., use of terrestrial vs. marine resources) and hence provide spatial information. In contrast, *δ*^15^N values show a stepwise increase of ~3 to 5 ‰ with trophic level [19]. Stable isotope values of tissues with different turnover rates provide dietary information over different temporal scales, as isotopic ratios are incorporated during the period of tissue formation [20]. For example, bone collagen integrates dietary information over several months or years, with short-term variation in diet having a minimal impact [21, 22]. Feathers have a shorter integration period; for instance, primary feathers of black kites *M*. *migrans* and common buzzards *Buteo buteo* are grown over 50–65 days [23]. Stable isotope analyses can be applied to museum specimens and archaeological and fossil bones to explore past diets [24–26], including those of raptors [27, 28], whereas samples for conventional diet analyses (e.g., stomach contents) are rarely preserved over long time periods.

In this study, we use stable isotope analyses of feather keratin and bone collagen of museum specimens (collected from the 1800s to 2010s) to compare the diets of reintroduced red kites and white-tailed eagles with individuals from the pre-extirpation populations in Scotland. Our sample collection incorporates periods of persecution, extirpation and reintroduction, and our objective was to determine whether these species shifted their diets since their reintroductions. Overall, our study examines the complex relationship between humans and scavenging raptors in Scotland, with a history of commensalism, persecution and species reintroduction.

## Materials and methods

### Sample collection

**Fig 1:**
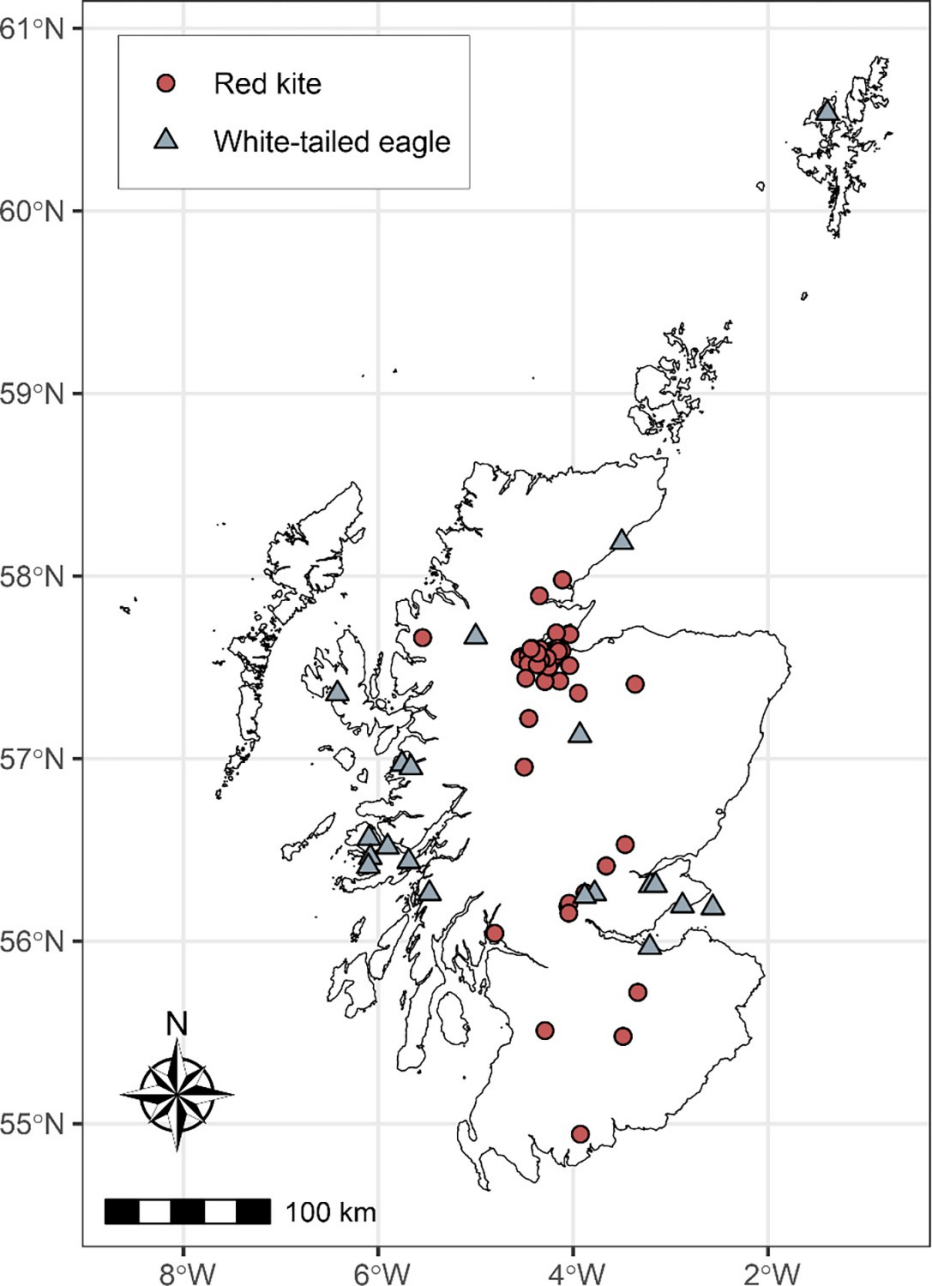
Collection locations of museum specimens analysed in this study throughout Scotland. Points indicate collection locations throughout Scotland of museum specimens of red kites *Milvus milvus* and white-tailed eagles *Halieaeetus* albicilla sampled for feathers (P1) and bone. Birds are categorised as pre- or post-reintroduction to Scotland based on collection dates. This map was created by the authors in R with the base map provided by Eurostat (GISCO, https://ec.europa.eu/eurostat/web/gisco). ©EuroGeographics for the administrative boundaries.

Fully grown primary feathers (P1) were sampled from museum specimens of 30 red kites and 10 white-tailed eagles, and rib bones from 36 red kites and 20 white-tailed eagles, respectively (Table 1). Accipitrid raptors replace their primaries sequentially from the innermost (P1) to outermost (P10) [29]. Adult red kites undergo a complete annual moult of their plumage (including the large flight feathers) between approximately April and October [30]. Primary feather moult of white-tailed eagles occurs as a wave moult pattern over multiple years, with P1 replaced at the beginning of each moult cycle, and moult is suspended from November to March [29, 31, 32]. All museum specimens included in our study had collection locations within Scotland (Fig 1) and were stored in the collections of National Museums Scotland (NMS; Edinburgh, UK). Our sample collection represents the entire specimen holdings of both taxa at NMS. Collection dates ranged from 1835–2017 and 1894–2013 for red kites and white-tailed eagles, respectively. We excluded from our analysis those samples with signs of pathological changes to bone, large-scale contamination (e.g., from paint) or without an exact date and geographical location. Birds were classified as pre- or post-reintroduction to Scotland according to their collection date (i.e., pre-1975 and 1989 for red kites and white-tailed eagles, respectively). No bone samples were available for pre-reintroduction red kites. Full details of each museum specimen are provided in S1 Table. No permits or considerations relating to protected species or animal welfare were needed to carry out this work, as all tissues sampled were from archival museum specimens, nor was any fieldwork conducted requiring access to privately-owned or protected land. Sampling was carried out under a loan arrangement (reference NS.OL.2021.20) from National Museums Scotland.

**Table 1:** Stable isotope values (‰) of carbon (*δ*^13^C) and nitrogen (*δ*^15^N) in tissues of reintroduced Scottish raptors. Stable isotope ratios were measured in feather keratin (P1) and bone collagen from museum specimens of red kites *Milvus milvus* and white-tailed eagles *Halieaeetus albicilla*. Data are grouped according to whether the specimen collection date corresponded to pre- or post-reintroduction to Britain. Values are means (± SDs) with minimum and maximum values in parentheses. SEAc = standard ellipse areas (‰^2^) corrected for small samples; TA = the total area (‰^2^) of convex hulls. *δ*^13^C values were corrected for the Suess effect. Analytical errors for bone collagen were ±0.07 ‰ and ±0.13 ‰ for *δ*^13^C and *δ*^15^N values, and ±0.09 ‰ and ±0.15 ‰ for feather keratin.

Species	Tissue	Period	*n*	*δ*^13^C (‰)	*δ*^15^N (‰)	TA (‰^2^)	SEAc (‰^2^)
Red kite	Bone collagen	Post-reintroduction	36	-21.5 ± 0.5 (-22.5 to -20.2)	9.4 ± 0.8 (7.2 to 10.8)	5.7	1.4
		Pre-reintroduction	-	-	-	-	-
	Feather keratin	Post-reintroduction	27	-23.3 ± 0.9 (-24.9 to -21.6)	8.8 ± 1.0 (6.0 to 10.5)	9.1	2.8
		Pre-reintroduction	3	-22.3 ± 0.3 (-22.6 to -22.0)	10.0 ± 0.9 (9.3 to 11.0)	-	-
White-tailed eagle	Bone collagen	Post-reintroduction	18	-18.9 ± 1.3 (-21.0 to -15.9)	11.2 ± 1.6 (8.6 to 14.2)	11.7	3.9
		Pre-reintroduction	2	-17.7 ± 3.3 (-20.0 to -15.4)	12.0 ± 2.2 (10.4 to 13.5)	-	-
	Feather keratin	Post-reintroduction	7	-20.6 ± 1.5 (-22.3 to -18.8)	10.8 ± 1.8 (8.8 to 13.3)	5.2	3.6
		Pre-reintroduction	3	-22.7 ± 1.5 (-24.3 to -21.4)	12.5 ± 2.7 (9.6 to 14.9)	-	-

### Stable isotope analysis

Subsamples of rib bones were removed using small shears to cut a section of bone from the sternal end, and subsamples were taken along the length of the feather (excluding the rachis) using a scalpel at 3-cm intervals and mean values were used in subsequent analyses. Lipid residues were extracted from the bones and feather samples were cleaned of surface contaminants using a chloroform:methanol solution (2:1 v:v), sonicated for 10 minutes and repeated until the solution was clear (minimum of 2 and mean of 3 repetitions). Samples were then rinsed three times in ultra-high-quality water (17 MΩ UHQ) in a sonicator bath and oven-dried at 35°C for 48 h. Cleaned and dried feather samples were then homogenised using stainless steel scissors. Bone collagen extraction was undertaken at the University of Reading following the method described by Longin [33] and modified according to Collins & Galley [34]. Bone samples were then demineralised in 0.5M HCl at room temperature until the bone was soft and effervescent reaction of the mineral component with acid ceased to occur (time varied for each sample, but was a minimum of 5 days). Following this, the demineralised samples were gelatinised in acidified water (pH 3) on a heating block at 72°C for 48 h. Following gelatinisation, the resulting solution was filtered using Ezee^TM^ filters to remove solid matter and frozen for 48 h, and then lyophilised to produce collagen. Bone collagen and feather samples were weighed in triplicate (~0.2 mg) into 8 x 5 mm tin capsules using a microbalance. Stable isotope ratios of carbon and nitrogen were measured using a continuous-flow isotope-ratio mass spectrometer coupled to a ThermoFisher™ DeltaV Advantage, fitted with an Isolink CNSOH Temperature Conversion Elemental Analyzer (TC/EA) and smart function at the University of Reading Chemical Analysis Facility. Results are expressed as *δ* values (‰) relative to the international references Vienna PeeDee Belemnite and atmospheric N_2_ (AIR) for carbon and nitrogen, respectively. Data were corrected for linearity and instrument drift (every five samples) and stretch corrected by analysing in-house (x3: MethR [*δ*^13^C -27.5 ‰; *δ*^15^N -4.1 ‰], Reading Fish Skin [*δ*^13^C -15.6 ‰; *δ*^15^N 14.0 ‰], Reading Porcine Gelatin [*δ*^13^C -21.5 ‰; *δ*^15^N 5.0 ‰]) and international standards (x5, including IAEA-CH-7 [*δ*^13^C -32.2 ‰], IAEA-601 [*δ*^13^C -28.8 ‰], USGS42 [*δ*^13^C -21.1 ‰; *δ*^15^N 8.1 ‰] and USGS43 [*δ*^13^C -21.3 ‰; *δ*^15^N 8.45 ‰]). Measured values of internal and international standards were also compared to expected values to calculate analytical error, which was <0.15 ‰ for *δ*^13^C and *δ*^15^N. C:N ratios of feather keratin samples included for study varied between 3.5 and 4 (mean 3.7), and bone collagen sample ratios were between 3.2 and 3.6 (mean 3.4), verifying satisfactory lipid removal. One red kite and one white-tailed eagle bone collagen sample were also prepared, but excluded from analysis and all sample counts as their C:N ratios were 4.0 and 3.9 respectively, outside of the range recommended by Guiry & Szpak [35].

### Data analysis

All data were analysed using Minitab® 21.4.2 and visualised using the ggplot2 package in R [36]. Anthropogenic releases of CO_2_ from the combustion of fossil fuels have resulted in declining atmospheric *δ*^13^C values, termed the ‘Suess effect’ [37]. Hence, because we analysed samples collected over several decades, we applied a year-specific correction factor to each raw *δ*^13^C value following Graven et al. [38], normalising each measurement to pre-1850 atmospheric conditions (measured values and correction factor are given in S1 Table). These corrections were also applied to data used for comparisons in our stable isotope biplot: northern fulmars *Fulmarus glacialis* [39] and European rabbits *Oryctolagus cuniculus* [40]. Prior to statistical analyses, stable isotope data were checked for the assumptions of normality of residuals and homogeneity of variances using Shapiro-Wilk and Levene’s tests, respectively. Among post-reintroduction birds, Mann-Whitney tests were used to test whether bone collagen and feather keratin *δ*^13^C and *δ*^15^N values differed between species. For the subset of birds for which both tissues were analysed, linear regressions were used to test whether feather *δ*^13^C and *δ*^15^N values were significantly related to those of bone collagen. These analyses were limited to red kites to remove confounding effects of taxa, as there were too few white-tailed eagle samples to allow for comparisons between the two. Statistical significance was assumed at α = 0.05 in all cases.

We used the Stable Isotope Bayesian Ellipses in R (SIBER) package in R to quantify the isotopic niches of reintroduced individuals [41]. The pre-reintroduction birds were not included in these analyses due to small sample sizes for each species-tissue group. We calculated the standard ellipse areas corrected for small sample sizes (SEAc) as a measure of the core isotopic niche and also the total area (TA) of the convex hull area, which encompasses all data points [40, 41]. This enabled us to quantify the trophic diversity of contemporary populations, compare isotopic niche width between species, and explore whether pre-reintroduction birds fell within these isotopic niche metrics. Full details of the six stable isotope metrics proposed by Layman et al. [41] are included in the Supporting Information (S2 Table).

## Results

**Fig 2:**
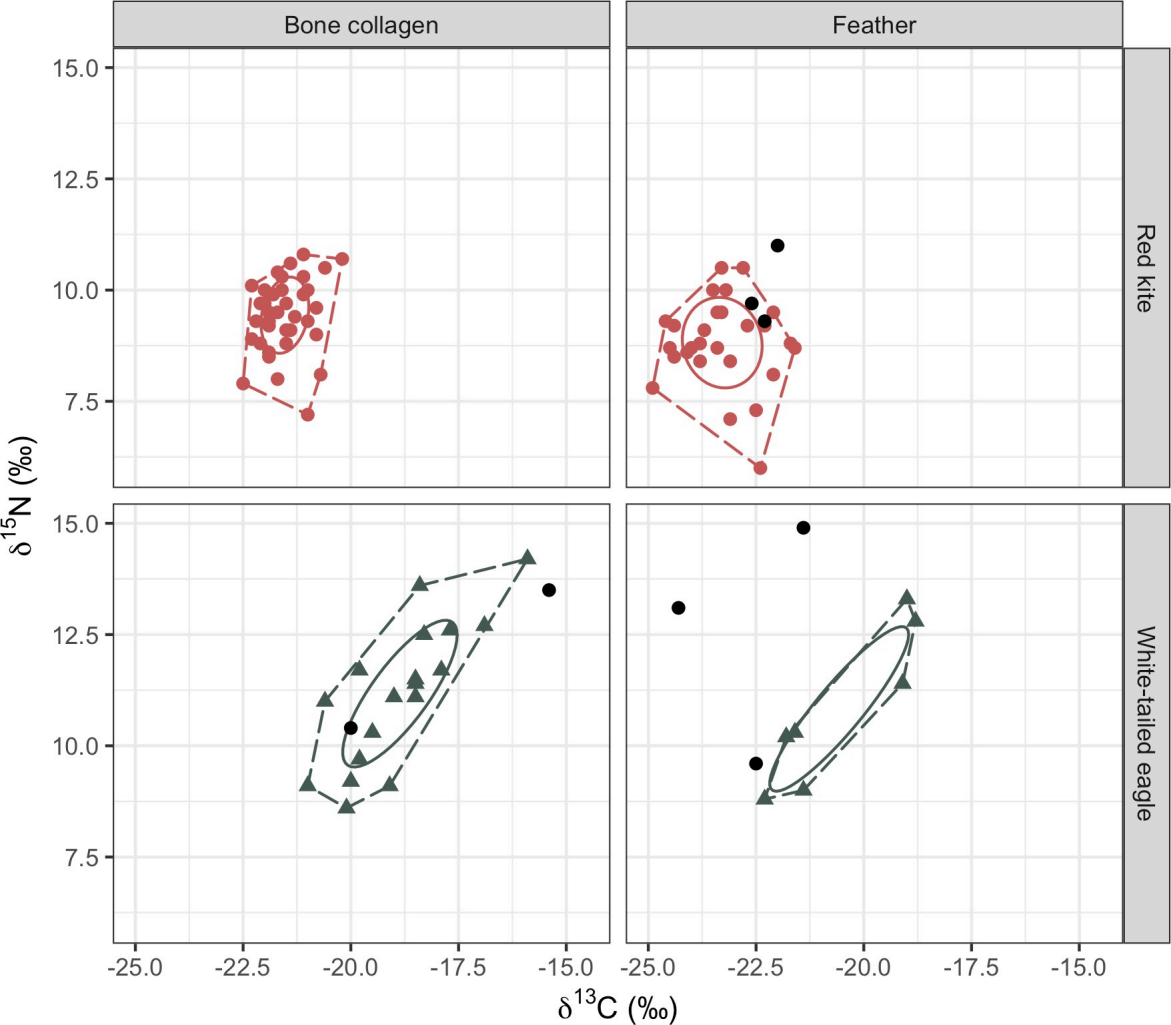
Stable isotope values of two tissues from reintroduced raptors in Scotland. Stable isotope values (‰) of carbon (*δ*^13^C) and nitrogen (*δ*^15^N) in feather keratin (P1) and bone collagen from museum specimens of red kite *Milvus milvus* and white-tailed eagle *Halieaeetus albicilla* collected throughout Scotland. Birds are categorised as pre- or post-reintroduction (filled and open circles, respectively) to Scotland based on collection dates. Dashed lines are total convex hulls for post-reintroduction birds. Solid lines indicate standard ellipse areas (‰^2^) corrected for small samples (SEAc) and dashed lines are the total areas (‰^2^) of convex hulls (TA).

**Fig 3:**
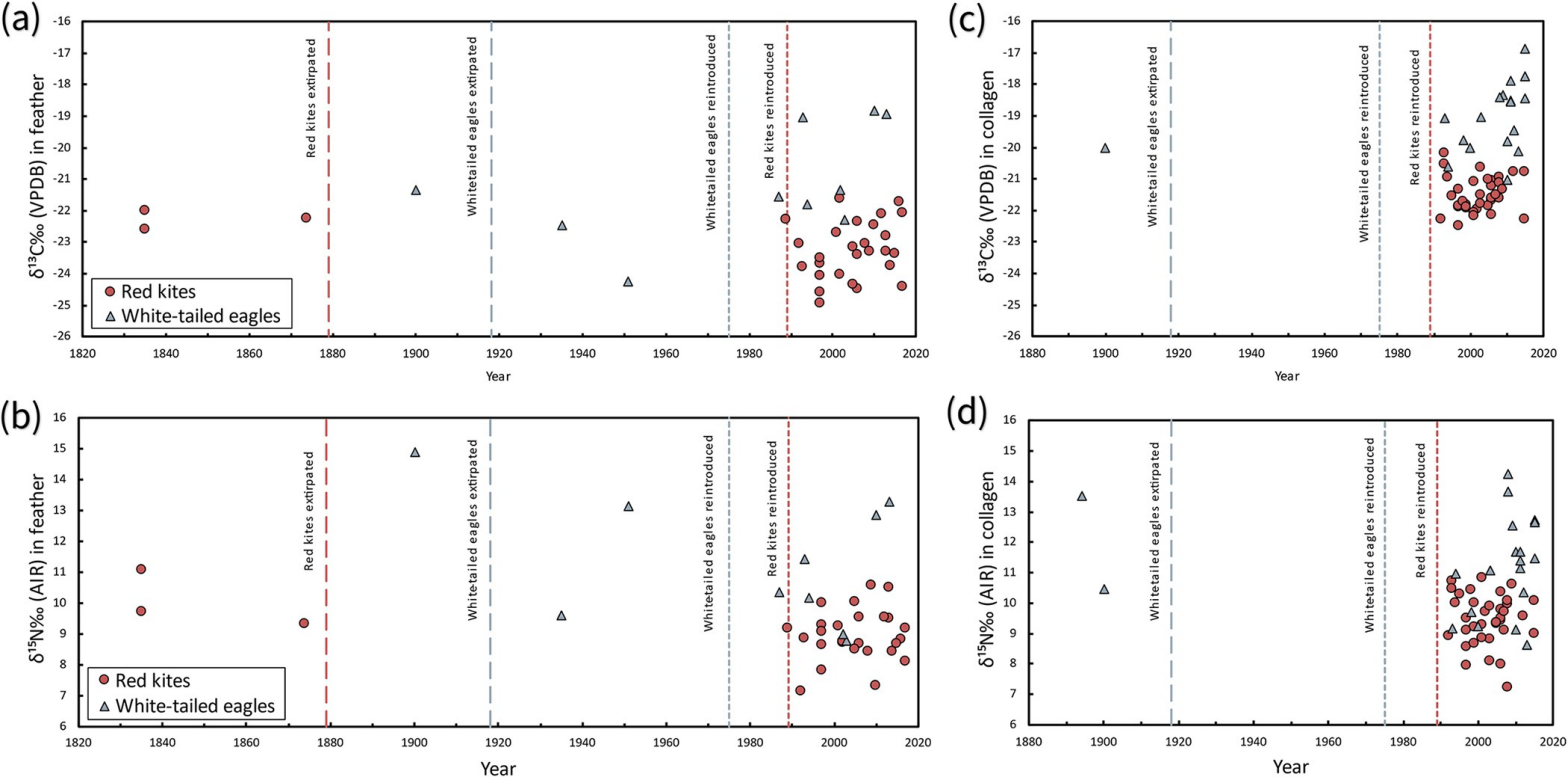
Temporal trends in stable isotope ratios of reintroduced raptors. Changes in stable isotope values (‰) of carbon (*δ*^13^C) and nitrogen (*δ*^15^N) over time, measured in feather keratin (**a, b**) and bone collagen (**c, d**) from red kites *Milvus milvus* and white-tailed eagles *Halieaeetus albicilla*, with *δ*^13^C values corrected for changes to atmospheric carbon after Graven et al. (2017).

**Fig 4:**
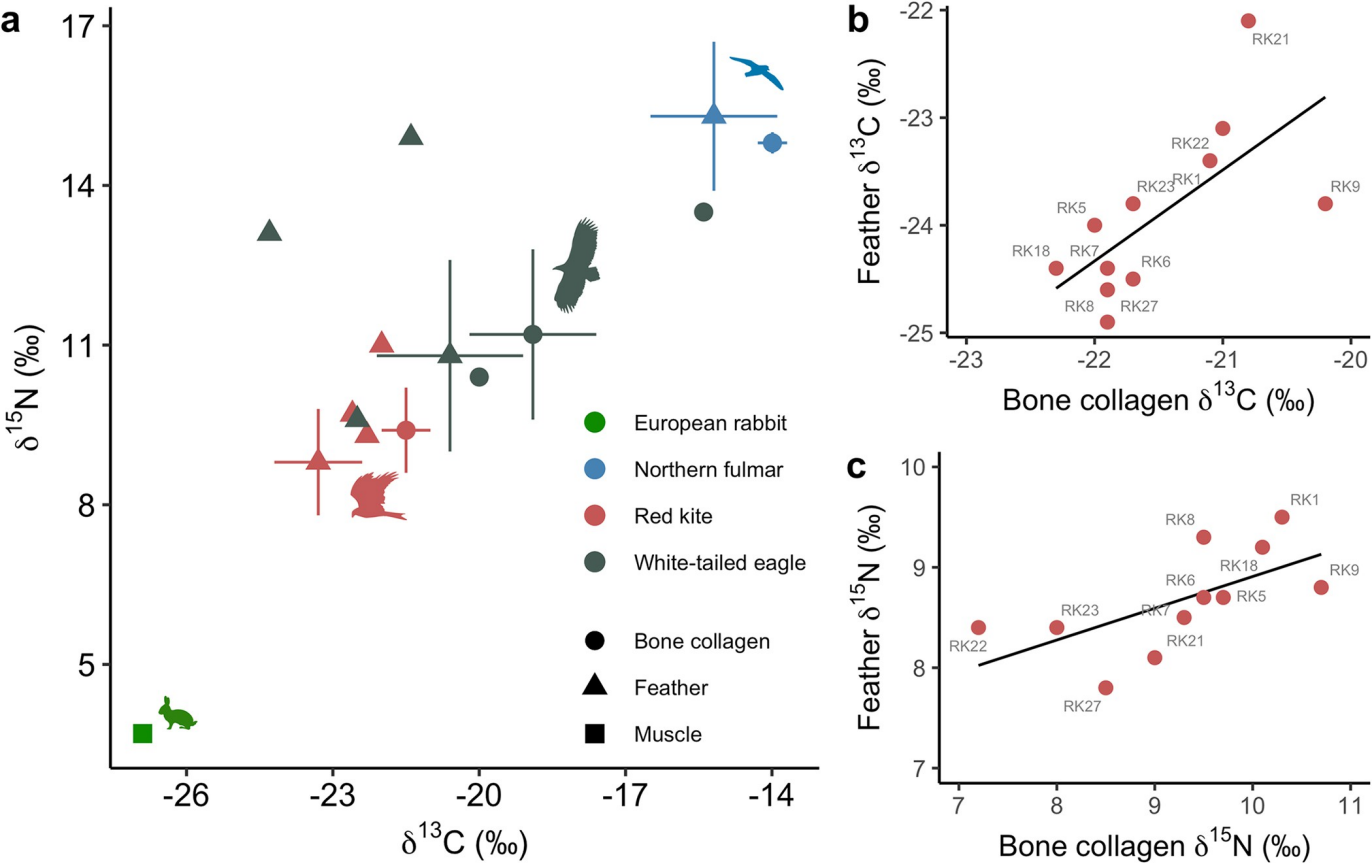
Comparing isotope values between tissues and comparative Scottish fauna. Carbon (*δ*^13^C) and nitrogen (*δ*^15^N) values (‰) for red kites *Milvus milvus* and white-tailed eagles *Haliaeetus albicilla* in bone collagen and feather keratin (**a**). Means and standard deviations for post-reintroduction populations are given, and pre-reintroduction birds are presented as single datapoints. Comparative isotope values for northern fulmars *Fulmarus glacialis* [39] and European rabbits *Oryctolagus cuniculus* [40] from Scotland are also given, representing marine predators and terrestrial prey respectively. Comparison between (**b**) *δ*^13^C (‰) and (**c**) *δ*^15^N (‰) values for bone collagen and feather from the same individual in red kites *Milvus milvus*. Solid lines are linear regressions.

Stable isotope ratios of carbon (*δ*^13^C) and nitrogen (*δ*^15^N) were measured in bone collagen and feather keratin of red kites (*n* = 37 and *n* = 30, respectively) and white-tailed eagles (*n* = 21 and *n* = 10) and are summarised in Table 1. Among post-reintroduction birds, bone collagen *δ*^13^C values of white-tailed eagles were significantly less negative and *δ*^15^N values were higher than those of red kites (Mann-Whitney tests, W = 676, p < 0.001 and W = 787, p < 0.001, respectively) (Table 1; Figs 2 and 3). Similarly, feather keratin *δ*^13^C values were significantly less negative and *δ*^15^N values were higher in reintroduced white-tailed eagles compared to red kites (W = 384, p < 0.001 and W = 408, p < 0.05). For all tissue-period combinations, standard deviations (SDs) associated with mean *δ*^13^C and *δ*^15^N values were larger for white-tailed eagles than red kites (Table 1 and Fig 2). There were significant positive relationships between the *δ*^13^C and *δ*^15^N values of feather keratin and bone collagen of red kites (linear regressions, F_1,9_ = 7.23, p < 0.05 and F_1,9_ = 6.07, p < 0.05) (Fig 4). For reintroduced white-tailed eagles, the TA was 11.7 ‰^2^ for bone collagen and 5.2 ‰^2^ for feather and SEAc values were 3.9 ‰^2^ for bone and 3.6 ‰^2^ for feather keratin. In red kites, total area was 5.7 ‰^2^ in bone collagen and 9.1‰^2^ for feather keratin, with SEAc values of 1.4‰^2^ and 2.8‰^2^ for collagen and feather keratin respectively. Feather keratin stable isotope values of two of three pre-reintroduction red kites in our sample collection were within the TA of the reintroduced birds, and one was outside (RK20) (Figs 2 and 3). Feather keratin stable isotope values of all (*n* = 3) pre-reintroduction white-tailed eagles were outside the TA of reintroduced birds, as were the bone collagen values of a single white-tailed eagle from Shetland circa 1894 (WTE3) (Figs 2 and 3).

## Discussion

### Diets of post-reintroduction birds

Our study found that, among reintroduced birds in Scotland, *δ*^13^C values were significantly less negative and *δ*^15^N values were significantly higher in feather keratin and bone collagen of white-tailed eagles compared to those of red kites. At the population level, these significant interspecific differences in stable isotope values are likely to be indicative of white-tailed eagles consuming more marine resources, especially high trophic level prey, compared to the terrestrial, lower trophic level prey of red kites in Scotland. Higher *δ*^15^N and more positive *δ*^13^C values together in carnivorous animals is typically interpreted in C4 biospheres as indicating marine foods in the diet (see e.g., northern fulmar data in Fig 4) [42], and conventional diet studies of white-tailed eagles contain marine resources in varying proportions [2, 43, 44]. All individuals of both taxa have high *δ*^15^N values for bone collagen (all ≥7.2 ‰), indicating long-term high-trophic level or predatory diets. In many cases, the birds were consuming prey that was itself already from a high trophic level: 31% of pre-reintroduction kites (*n* = 11/36) and 72% of pre-reintroduction eagles (*n* = 13/18) had collagen *δ*^15^N values >10 ‰. Trophic discrimination factors are not known for these species; however, in ring-billed gulls *Larus delawarensis*, collagen to diet fractionation was +3.1 ‰ for *δ*^15^N and +2.6 ‰ for *δ*^13^C, and feather to diet +3.0 ‰ and +0.2 ‰ respectively [45]. Mean values across all tissues calculated by Post [46] match this, which found a mean diet-tissue fractionation factor of +3.4 ‰ for nitrogen. Using these values, the average diet prey items have *δ*^15^N values of ~7 ‰ or higher. As herbivores in terrestrial ecosystems typically have values around 4–6 ‰ (for instance, rabbits, Fig 4), this indicates that birds may be incorporating higher-trophic-level food items. For the white-tailed eagles the most likely prey items include high-trophic-level predatory fishes of large body size, and for red kites this may include scavenged prey of omnivores, including badgers *Meles meles* and red foxes *Vulpes vulpes*, especially in areas where such food items are abundant as roadkill or killed as pests. This was the case for many of the eagles, with 78% (*n* = 14/18) having collagen *δ*^13^C values less negative than -20.0 ‰, compared to red kites where the least negative *δ*^13^C value was -20.2 ‰.

Our isotopic data from reintroduced white-tailed eagles agree with conventional diet studies in Scotland. Madders & Marquiss (2003) found that white-tailed eagles incorporated more seabirds and fishes into their diets, which were also more diverse, than golden eagles, *Aquila chrysaetos*, occupying the same areas in Scotland [47]. When terrestrial food items were present, it was typically deer and sheep *Ovis aries* (as scavenged prey) rather than lagomorphs, which were found more frequently in association with golden eagles [47]. A later Scottish study [48], using the same methods, echoed these findings. The primary food sources for the white-tailed eagle were waterbirds, sheep, and fishes, compared to the gamebirds and lagomorphs exploited by the golden eagle [48]. Marquiss et al. (2003) [49] investigated the presence of lamb specifically in the diet of Scottish eagles, finding that except for a single pair who seemed to specialise hunting live lambs, eagles were otherwise only eating scavenged lambs in small quantities. Reid et al. (2023) found that birds (primarily seabirds) comprised 67% of white-tailed eagle diets; marine fishes were the next most important prey, although they may have been underrepresented in nest remains [2]. They found limited evidence for specialisation, with most pairs consuming a diverse range of prey, with large variation between territories, such as more terrestrial and wetland prey and sites further inland, but with overall consistency between years [2]. Our isotope data also suggest that marine items are a significant part of the diets of the reintroduced white-tailed eagle population in Scotland. The white-tailed eagles with lower *δ*^15^N and more negative *δ*^13^C values in our study may be those who are consistently exploiting large mammal carcasses through scavenging.

The red kites in this study have stable isotope values consistent with a terrestrial, carnivorous diet, with individual birds consuming prey of varying trophic levels (or mixtures thereof). Their known diets in Scotland are varied, with a large number of different prey items observed in traditional ecological studies. Five years after red kites were reintroduced to Scotland, rabbits were the most numerous dietary item throughout the year (isotope values for rabbits [40] are included in Fig 4 as an example of possible prey values); however, kites fed on sheep carrion, earthworms and rodents in winter, and on corvids in summer [50]. Following reintroduction to the English midlands, rabbits and earthworms were the most frequently observed prey items in pellets and field observations of red kites, and most of the diet was assumed to be scavenged rather than actively predated [51]. Similar results were obtained in the north-east of England, where pellet analysis revealed a large proportion of earthworms and small birds, with larger mammals eaten as carrion, such as lagomorphs and roe deer *Capreolus capreolus*, although this study only focused on the winter period when other food sources are scarce [52]. The diversity in food items identified by previous authors is reflected in the varied isotope values of the Scottish population, with a mixture of high- and lower- trophic-level prey items, although unlike the white-tailed eagle, they are restricted to terrestrial ecosystems. Even though high levels of variation in short-term diets can be expected from opportunistic predators when foodstuffs become available, their diets are highly generalised and likely represent a mixture of the most consistently abundant prey resources in their habitat. Anthropogenic foods are often incorporated into modern kite diets. Red kites in Wales before their reintroduction to the rest of the country were found to feed mainly on scavenged sheep, but authors found evidence for anthropogenic forage in the form of butcher’s wrappings and cellophane packaging in pellets [53]. Deliberate garden feeding of red kites has not been reported as regular practice in rural Scotland, but is known to occur elsewhere in their reintroduced range: survey data from urban Reading, where garden feeding of kites is practised by up to 1 in 20 households, suggests this could support between 142–320 individuals [16]. Carter and Grice [51] highlighted the role of anthropogenic food in their midlands study, suggesting that much of the scavenged food consumed by kites may come from roadkill and pest-control killing, concluding that “it is somewhat ironic that a species almost wiped out in Britain as a result of human persecution is now dependent on human support, either directly or indirectly, for much of its food”. In Scotland, especially the rural areas where kites are found, anthropogenic food contributions likely include scavenged livestock waste from sheep farming and occasional supplementary feeding at feeding stations, which typically feed butchery waste.

### Diet breadth

In our study, for both tissues, *δ*^13^C and *δ*^15^N values of white-tailed eagles were more variable (i.e., greater SDs and SEAc) than those of red kites (Fig 4). As well as an indication of diet as a whole in both taxa, assessing the variability of isotope values between individual birds can help understand niche width within the population of each taxon. Typically, populations with a wide niche (generalists) would be expected to have a larger isotopic range between individuals than those with a small niche (specialists; [54]). The reintroduced white-tailed eagles had a large range between the lowest and highest bone collagen isotope values for both *δ*^15^N, indicating carnivorous diets with varying levels of high-trophic level prey, and *δ*^13^C, suggesting diets with varying degrees of marine, freshwater, and terrestrial sources. The wide isotopic range of the white-tailed eagles is compatible with their known ecological niche that includes fishes, waterbirds, and terrestrial mammals, including some food items with very high *δ*^15^N values, such as seals [55], which average 17.0 ± 2.1‰ across a global distribution [56]. Red kites are known to be generalists and opportunists in much of their range [51, 57], but variation among reintroduced individuals was more constrained compared to the white-tailed eagles for both *δ*^15^N and *δ*^13^C values. This suggests that individual birds may have exploited a range of food resources, or alternatively that they exploited a wide range of foods with an equifinal isotopic output. As red kites are not known to consume fish in substantial quantities. their smaller niche width is unsurprising, as even if a wide variety of prey items is exploited, it is limited to the narrower isotopic range of the terrestrial biosphere. Juvenile white-tailed eagles are known to forage over wide ranges before reaching breeding age [58], and it is possible that some variation between individuals relates to feeding in habitats with different *δ*^13^C baselines, although these were not expected to vary significantly within the latitude range of Scotland’s mainland and islands [59]. The variation in isotope values between the two tissues cannot be used to calculate fractionation between the bone and feathers, as it may also vary due to shifts in diet through time, with the bone giving a long-term dietary average, and the feather a shorter-term dietary signal. Despite this, as nearly all birds have less negative values for *δ*^13^C in bone collagen, these results indicate a differing fractionation of ^13^C in the skeleton compared to feather in raptors. This was also seen in northern fulmars *Fulmarus glacialis*, where bone collagen averaged *δ*^13^C values of -15.3 ‰ compared to -16.5 ‰ in wing feathers of birds eating consistent diets (Fig 4) [39]. The relationship between bone and feather *δ*^15^N was less consistent, although much of this was due to RK22, which had the lowest collagen *δ*^15^N values, (7.2 ‰) and may have represented a period of dietary variance that was not reflected in the feather samples, which captured more recent diet. The relationship between feather and collagen *δ*^15^N shows high correlation (Fig 4). These results suggest that although biological fractionation between tissues may occur, it was complicated by the effects of short-term dietary variation.

### Stable isotope insights from other populations

No previous studies with published *δ*^13^C and *δ*^15^N values from any tissue in red kites were available for comparison with this dataset, but stable isotope values from eagles in other regions were used to contextualise our results from the Scottish population. Stable isotope values of livers and muscle from white-tailed eagles in Germany, Finland, and Greenland indicate that marine and terrestrial resources varied between these populations, with a greater component of terrestrial scavenging in German birds [44]. There was also a high degree of variation among individual birds and even within temporally distinct measurements from the same birds in the German population [44]. Our results from the Scottish white-tailed eagles seem superficially similar to this German group, as isotope values within our study group were varied, with different dietary patterns between individuals, reflecting varying degrees of marine dominance in the diet, which may relate to location, prey availability or individual specialisation [60]. Mean liver *δ*^13^C and *δ*^15^N values of white-tailed eagles in northern Germany were -24.7‰ and 12.4‰, respectively, and were interpreted as reflecting a diet comprising more fishes and fish-eating waterbirds than mammal protein [61]. This is likely the case for the Scottish white-tailed eagles with the highest *δ*^15^N values in our study, which are closest to the northern fulmar, (Fig 4), an exclusively marine predator [39].

The variability in white-tailed eagle diet is particularly significant as it may be influenced by the availability of foodstuffs owing to human presence. In ecological studies of white-tailed eagles, aquatic foodstuffs form the majority of the diet, but when terrestrial foods are added they are often scavenged and may also be anthropogenic in origin. These include livestock and introduced game birds, as well as wild game, such as red deer *Cervus elaphus* from managed parks, carcasses from natural mortalities and remnants from gralloching. For some of these Scottish eagles with lower values for *δ*^15^N and more negative values for *δ*^13^C, which plot close to the range of the red kites, these terrestrial food sources may have been particularly important. WTE5 is the most extreme example of these birds with values of -21.0‰ and 9.1‰, and was recovered from Perthshire near railway lines, possibly following a collision. Incorporating anthropogenic foods may also include items from feeding stations and other human provision, as discussed for red kites above. There are usually assumed to be supplementary items and not contribute significantly to overall dietary intake, but it is also possible that some of the more terrestrial diets observed in the Scottish population indicates a greater dependence on these items.

### Evidence for changing diets?

Our analyses of museum specimens permitted the examination of long-term differences in trophic ecology of our two study species before and after their reintroductions to Scotland. One white-tailed eagle bone collagen sample from a bird pre-dating the reintroduction period (WTE3) had isotopic values outside the TA of post-reintroduction birds, although it was from a bird collected in Shetland in the autumn of 1894. White-tailed eagles have not yet fully colonised Shetland, and visit but do not breed or reside there, so none could be sampled for stable isotope analysis. Direct comparisons of changes to dietary isotope niche through time within this region were not possible, but it is possible that at this edge of their Scottish range their diet would be similarly distinct from the modern-day reintroduced birds elsewhere in Scotland. Marine mammals were also hunted onto beaches in Shetland at this time [62], which could have provided carrion food sources with higher *δ*^15^N and more positive *δ*^13^C values at more regular intervals than other parts of Scotland where beached cetaceans occurred less frequently. For feather keratin, all three pre-reintroduction eagles were outside of the TA of modern eagle values, which could reflect a different dietary strategy in the pre-reintroduction population, but this may also be due to the small number of feather samples (modern population *n* = 6). White-tailed eagles also have not reconquered all their former range in Scotland today, and the precise location of many of the historic museum specimen eagles was not known, so it could reflect a region of their past range that was isotopically distinct, as is the case for the Shetland bird WTE3. Alternatively, these samples seem to have a greater proportion of marine dietary components or other high-trophic level prey than the modern TA, which could reflect changing prey availability, with greater access to high-trophic level items and fewer opportunities for terrestrial scavenging. In red kites, the single pre-reintroduction feather sample that fell out of the modern TA was from Shandon, Argyll, circa 1835, although another pre-reintroduction bird also originated from the same location and time period that was within the modern TA. This may indicate individual specialisation on a prey item unavailable to modern birds, or more general shifts in prey availability exploited differently by the two Shandon birds at different times. Although the remaining historic red kites were within modern ranges for *δ*^13^C and *δ*^15^N values, they were towards the higher end of the modern range. This suggests that the foraging niche of red kites has remained largely consistent in pre- and post-reintroduction populations, but that historic kites likely consumed high-trophic-level prey items in greater numbers than many modern kites. It is unclear whether this reflects a greater abundance of high-trophic level prey previously, or a greater abundance of low-trophic level prey currently. Crucially, large numbers of wild rabbits were established at a later date in the Highlands compared to other regions of Britain: they were first seen on Wester Ross only in 1851 [63], so they were unlikely to have been a key prey item for many Highland birds in this period. However, although rabbit populations are a well-known ecological driver, it is unclear how much this is contributing to the changing diets of birds of prey. There are few conventional diet data for pre-reintroduction birds of either species, although the analyses of kites in Wales prior to their reintroduction to the rest of the country found that the main food item was scavenging of farmed sheep [53].

## Conclusion

Our study is the first stable isotope study of red kites and white-tailed eagles in Britain and fills a gap in the current understanding of the dietary niches of these generalist raptors, whose population success has been strongly linked with their utilisation of anthropogenic foods. Previous ecological studies have provided a basis for dietary diversity in these taxa, highlighting a wide variety of potential prey items. However, the study of diets through the seasonal remains of prey and pellets has inherent biases [64] and can only provide insights into the diets of modern birds. This study traces the dietary patterns of historic and modern birds, and reveals a wide dietary niche at a population level for both taxa, with especially broad foraging niches in white-tailed eagles. The white-tailed eagle diets revealed through *δ*^13^C and *δ*^15^N isotopic ratios indicate that the birds were consuming varying amounts of high-trophic-level marine and terrestrial protein. For historic specimens from the pre-reintroduction era, isotope values for many birds fall outside the ranges seen in eagle post-reintroduction, although it is unclear whether this apparent change in diet reflects shifts in distribution, prey availability, or is simply an artifact of small sample size. For red kites nearly all pre-reintroduction individuals fell within the ranges of modern birds, suggesting that their prey base was less likely to have altered substantially over the period spanning their extirpation and reintroduction to Scotland.

## Supporting information

S1 Table Full sample data for red kite *Milvus milvus* and white-tailed eagle *Halieaeetus albicilla* bone collagen and feather keratin samples analysed. All samples from specimens in the collections of National Museums Scotland, with date of collection are given. Dates correspond to when birds were found dead, which in some cases is based on contextual information in museum acquisition documentation. Find dates in some cases may be later than the date of death of the bird. Mean values for *δ*^15^N in feather keratin and bone collagen are presented along with mean measured *δ*^13^C values, correction for Suess atmospheric changes (after Graven et al., 2017) and adjusted *δ*^13^C values corrected to pre-1850 atmospheric conditions are given. Quality control parameters including weight percentages for carbon (wt%C), nitrogen (wt%N), atomic ratio (C:N_atomic_) are also given.
(DOCX)

S2 Table Layman’s metrics calculated for stable isotope values (‰) of carbon (δ^13^C) and nitrogen (δ^15^N) of bone collagen and feather keratin of red kites *Milvus milvus* and white-tailed eagles *Halieaeetus albicilla*. Abbreviations are: CR = range in δ^13^C values; NR: = range in δ^15^N values; TA = total area of the convex hull area that encompasses all data points (‰^2^); CD = mean distance to centroid; NND = mean nearest neighbour distance; SDNND = standard deviation of nearest neighbour distance. All birds were sampled post-reintroduction.
(DOCX)
