# Supplementary material for: The return of raptors to Scotland’s skies: Investigating the diets of reintroduced red kites and white-tailed eagles using stable isotopes
Source: PLoS One. 2025 Jan 8;20(1):e0315945. doi: 10.1371/journal.pone.0315945 (PMC11709231; doi:10.1371/journal.pone.0315945)
Supplement: S1 Table — All samples from specimens in the collections of National Museums Scotland, with date of collection are given. Dates correspond to when birds were found dead, which in some cases is based on contextual information in museum acquisition documentation. Find dates in some cases may be later than the date of death of the bird. Mean values for δ15N in feather keratin and bone collagen are presented along with mean measured δ13C values, correction for Suess atmospheric changes (after Graven et al., 2017) and adjusted δ13C values corrected to pre-1850 atmospheric conditions are given. Quality control parameters including weight percentages for carbon (wt%C), nitrogen (wt%N), atomic ratio (C:Natomic) are also given. (DOCX) [file pone.0315945.s001.docx]

**Table S1. Full sample data for red kite *Milvus milvus* and white-tailed eagle *Halieaeetus albicilla* bone collagen and feather keratin samples analysed.** All samples from specimens in the collections of National Museums Scotland, with date of collection are given. Dates correspond to when birds were found dead, which in some cases is based on contextual information in museum acquisition documentation. Find dates in some cases may be later than the date of death of the bird. Mean values for *δ*^15^N in feather keratin and bone collagen are presented along with mean measured *δ*^13^C values, correction for Suess atmospheric changes (after Graven et al., 2017) and adjusted *δ*^13^C values corrected to pre-1850 atmospheric conditions are given. Quality control parameters including weight percentages for carbon (wt%C), nitrogen (wt%N), atomic ratio (C:N_atomic_) are also given.

| Sample | NMS no. | Year | Period | Taxon | Tissue | Mean measured *δ*^13^C (‰) | Suess correction factor | Corrected δ13C | Mean *δ*^15^N (‰) | wt% C | wt% N | C:N^atomic^ |
| --- | --- | --- | --- | --- | --- | --- | --- | --- | --- | --- | --- | --- |
| RK 1 | GN90894 | 2006 | Post-reintroduction | Red kite | Feather keratin | -25.0 | 1.63 | -23.4 | 9.5 | 44.9 | 14.8 | 3.5 |
| RK 5 | GN51864 | 2002 | Post-reintroduction | Red kite | Feather keratin | -25.5 | 1.5 | -24.0 | 8.7 | 43.8 | 14.7 | 3.5 |
| RK 6 | NMS.Z.2011.105.2 | 2006 | Post-reintroduction | Red kite | Feather keratin | -26.1 | 1.63 | -24.5 | 8.7 | 44.9 | 14.9 | 3.5 |
| RK 7 | NMS.Z.2011.112.3 | 2005 | Post-reintroduction | Red kite | Feather keratin | -26.0 | 1.6 | -24.4 | 8.5 | 43.2 | 14.3 | 3.5 |
| RK 8 | NMS.Z.1999.32.7 | 1997 | Post-reintroduction | Red kite | Feather keratin | -26.0 | 1.37 | -24.6 | 9.3 | 44.5 | 14.5 | 3.6 |
| RK 9 | NMS.Z.1994.9.2 | 1993 | Post-reintroduction | Red kite | Feather keratin | -25.0 | 1.24 | -23.8 | 8.8 | 45.1 | 14.8 | 3.6 |
| RK 11 | NMS.Z.1888.84.148 | 1874 | Pre-reintroduction | Red kite | Feather keratin | -22.3 | 0.06 | -22.3 | 9.3 | 45.1 | 14.4 | 3.7 |
| RK 12 | NMS.Z.1880.34 | 1835 | Pre-reintroduction | Red kite | Feather keratin | -22.6 | 0 | -22.6 | 9.7 | 45.8 | 14.3 | 3.7 |
| RK 13 | NH80987/28287 | 2016 | Post-reintroduction | Red kite | Feather keratin | -23.6 | 1.83 | -21.7 | 8.8 | 45.6 | 14.7 | 3.6 |
| RK 14 | NMS.Z.1999.82.3 | 1997 | Post-reintroduction | Red kite | Feather keratin | -25.5 | 1.37 | -24.1 | 8.6 | 44.6 | 14.3 | 3.6 |
| RK 15 | SR86.18 | 2015 | Post-reintroduction | Red kite | Feather keratin | -25.2 | 1.83 | -23.4 | 8.7 | 46.2 | 14.6 | 3.7 |
| RK 16 | GC08364 | 2009 | Post-reintroduction | Red kite | Feather keratin | -25.0 | 1.68 | -23.3 | 10.5 | 46.3 | 14.6 | 3.7 |
| RK 17 | GR42383 | 2013 | Post-reintroduction | Red kite | Feather keratin | -25.1 | 1.78 | -23.3 | 9.5 | 46.1 | 14.7 | 3.7 |
| RK 18 | SR16.17 | *2017* | Post-reintroduction | Red kite | Feather keratin | -26.3 | 1.83 | -24.4 | 9.2 | 46.2 | 14.5 | 3.7 |
| RK 19 | NMS.Z.1880.34 | 2005 | Post-reintroduction | Red kite | Feather keratin | -24.8 | 1.6 | -23.2 | 10.0 | 46.7 | 14.8 | 3.7 |
| RK 20 | 030.36.05 | 1835 | Pre-reintroduction | Red kite | Feather keratin | -22.0 | 0 | -22.0 | 11.0 | 46.4 | 14.5 | 3.7 |
| RK 21 | SR24.17 | 2017 | Post-reintroduction | Red kite | Feather keratin | -23.9 | 1.83 | -22.1 | 8.1 | 46.5 | 14.9 | 3.7 |
| RK 22 | SR71.17 | 2008 | Post-reintroduction | Red kite | Feather keratin | -24.8 | 1.67 | -23.1 | 8.4 | 45.9 | 14.4 | 3.7 |
| RK 23 | GH261.14 | 2014 | Post-reintroduction | Red kite | Feather keratin | -25.6 | 1.81 | -23.8 | 8.4 | 46.7 | 14.8 | 3.7 |
| RK 24 | NMS.Z.1994.9.5 | 1992 | Post-reintroduction | Red kite | Feather keratin | -24.3 | 1.24 | -23.1 | 7.1 | 46.8 | 14.6 | 3.7 |
| RK 25 | DB89.09 | 2006 | Post-reintroduction | Red kite | Feather keratin | -24.0 | 1.63 | -22.4 | 6.0 | 46.9 | 14.7 | 3.7 |
| RK 26 | GF53474 | 2013 | Post-reintroduction | Red kite | Feather keratin | -24.6 | 1.78 | -22.8 | 10.5 | 47.2 | 14.3 | 3.9 |
| RK 27 | NMS.Z.1999.82.2 | 1997 | Post-reintroduction | Red kite | Feather keratin | -26.3 | 1.37 | -24.9 | 7.8 | 46.3 | 14.3 | 3.8 |
| RK 28 | GN51861 | 2002 | Post-reintroduction | Red kite | Feather keratin | -23.1 | 1.5 | -21.6 | 8.7 | 45.8 | 14.0 | 3.8 |
| RK 29 | NMS.Z.1996.18 | 1989 | Post-reintroduction | Red kite | Feather keratin | -23.5 | 1.23 | -22.3 | 9.2 | 46.7 | 14.6 | 3.7 |
| RK 30 | DB5.02 | 1997 | Post-reintroduction | Red kite | Feather keratin | -25.1 | 1.37 | -23.7 | 9.1 | 46.9 | 14.5 | 3.8 |
| RK 31 | NMS.Z.2012.128.2 | 2012 | Post-reintroduction | Red kite | Feather keratin | -23.9 | 1.75 | -22.1 | 9.5 | 46.6 | 14.3 | 3.8 |
| RK 32 | DB11.02 | 2001 | Post-reintroduction | Red kite | Feather keratin | -24.2 | 1.46 | -22.7 | 9.2 | 47.0 | 14.5 | 3.8 |
| RK 33 | DB6.02 | 1997 | Post-reintroduction | Red kite | Feather keratin | -24.9 | 1.37 | -23.5 | 10.0 | 46.9 | 14.4 | 3.8 |
| RK 57 | PS 351/04 - 031/17/2 | *2010* | Post-reintroduction | Red kite | Feather keratin | -24.2 | 1.7 | -22.5 | 7.3 | 46.7 | 14.3 | 3.8 |
| WTE 1 | NMS.Z.2011.65.2 | 2003 | Post-reintroduction | White-tailed eagle | Feather keratin | -23.8 | 1.54 | -22.3 | 8.8 | 45.9 | 14.7 | 3.7 |
| WTE 2 | NMS.Z.1999.112.2 | 1993 | Post-reintroduction | White-tailed eagle | Feather keratin | -20.3 | 1.24 | -19.1 | 11.4 | 45.9 | 14.8 | 3.6 |
| WTE 4 | B55194 | 1994 | Post-reintroduction | White-tailed eagle | Feather keratin | -23.1 | 1.27 | -21.8 | 10.2 | 44.8 | 14.4 | 3.6 |
| WTE 9 | ZZ1975 | 2010 | Post-reintroduction | White-tailed eagle | Feather keratin | -20.5 | 1.7 | -18.8 | 12.8 | 44.5 | 14.5 | 3.6 |
| WTE 10 |  | 1935 | Pre-reintroduction | White-tailed eagle | Feather keratin | -22.8 | 0.31 | -22.5 | 9.6 | 45.8 | 14.7 | 3.6 |
| WTE 11 | NMS.Z.1991.121.1 | 1987 | Post-reintroduction | White-tailed eagle | Feather keratin | -22.7 | 1.14 | -21.6 | 10.3 | 45.9 | 14.7 | 3.6 |
| WTE 12 |  | 1951 | Pre-reintroduction | White-tailed eagle | Feather keratin | -24.6 | 0.37 | -24.3 | 13.1 | 44.5 | 14.1 | 3.7 |
| WTE 13 |  | 1900 | Pre-reintroduction | White-tailed eagle | Feather keratin | -21.5 | 0.12 | -21.4 | 14.9 | 45.9 | 14.6 | 3.7 |
| WTE 14 | NMS.Z.2013.32.1 | 2013 | Post-reintroduction | White-tailed eagle | Feather keratin | -20.7 | 1.78 | -19.0 | 13.3 | 44.9 | 14.7 | 3.6 |
| WTE 15 | SASA 02068 | 2002 | Post-reintroduction | White-tailed eagle | Feather keratin | -22.9 | 1.5 | -21.4 | 9.0 | 44.5 | 14.7 | 3.5 |
| RK1 | GN90894 | 2006 | Post-reintroduction | Red kite | Bone collagen | -22.7 | 1.63 | -21.1 | 10.3 | 50.4 | 17.9 | 3.3 |
| RK2 | SASA 06048 | 2006 | Post-reintroduction | Red kite | Bone collagen | -22.9 | 1.63 | -21.3 | 9.4 | 50.7 | 17.9 | 3.3 |
| RK3 | GF28873 | 2012 | Post-reintroduction | Red kite | Bone collagen | -22.6 | 1.75 | -20.8 | 9.6 | 51.1 | 18.0 | 3.3 |
| RK4 | PS345.04 | 2001 | Post-reintroduction | Red kite | Bone collagen | -22.6 | 1.46 | -21.1 | 10.8 | 51.0 | 17.5 | 3.4 |
| RK5 | GN51864 | 2002 | Post-reintroduction | Red kite | Bone collagen | -23.5 | 1.5 | -22.0 | 9.7 | 51.8 | 18.0 | 3.4 |
| RK6 | NMS.Z.2011.105.2 | 2006 | Post-reintroduction | Red kite | Bone collagen | -23.3 | 1.63 | -21.7 | 9.5 | 51.2 | 18.3 | 3.3 |
| RK7 | NMS.Z.2011.112.3 | 2005 | Post-reintroduction | Red kite | Bone collagen | -23.5 | 1.6 | -21.9 | 9.3 | 51.6 | 17.7 | 3.4 |
| RK8 | NMS.Z.1999.32.7 | 1997 | Post-reintroduction | Red kite | Bone collagen | -23.3 | 1.37 | -21.9 | 9.5 | 50.5 | 18.1 | 3.3 |
| RK9 | NMS.Z.1994.9.2 | 1993 | Post-reintroduction | Red kite | Bone collagen | -21.5 | 1.24 | -20.2 | 10.7 | 50.7 | 17.2 | 3.4 |
| RK18 | SR16.17 | 2015 | Post-reintroduction | Red kite | Bone collagen | -24.1 | 1.83 | -22.3 | 10.1 | 51.0 | 18.1 | 3.3 |
| RK21 | SR24.17 | 2015 | Post-reintroduction | Red kite | Bone collagen | -22.6 | 1.83 | -20.8 | 9.0 | 50.6 | 17.3 | 3.4 |
| RK22 | SR71.17 | 2008 | Post-reintroduction | Red kite | Bone collagen | -22.6 | 1.67 | -21.0 | 7.2 | 51.1 | 17.2 | 3.5 |
| RK23 | GH261.14 | 2006 | Post-reintroduction | Red kite | Bone collagen | -23.3 | 1.63 | -21.7 | 8.0 | 51.3 | 17.2 | 3.5 |
| RK27 | NMS.Z.1999.82.2 | 1997 | Post-reintroduction | Red kite | Bone collagen | -23.3 | 1.37 | -21.9 | 8.5 | 50.3 | 17.7 | 3.3 |
| RK34 | GN51782 | 2003 | Post-reintroduction | Red kite | Bone collagen | -22.2 | 1.54 | -20.7 | 8.1 | 48.2 | 16.2 | 3.5 |
| RK35 | GN69170 | 2007 | Post-reintroduction | Red kite | Bone collagen | -23.2 | 1.65 | -21.5 | 9.1 | 50.5 | 17.6 | 3.4 |
| RK36 | GK21396 | 2008 | Post-reintroduction | Red kite | Bone collagen | -22.8 | 1.67 | -21.1 | 9.9 | 51.0 | 17.7 | 3.4 |
| RK37 | GC76382 | 2008 | Post-reintroduction | Red kite | Bone collagen | -23.3 | 1.67 | -21.6 | 10.0 | 50.9 | 17.5 | 3.4 |
| RK38 | GH79.10 | 2009 | Post-reintroduction | Red kite | Bone collagen | -23.0 | 1.68 | -21.4 | 10.6 | 52.3 | 17.5 | 3.5 |
| RK39 | NMS.Z.1999.82.5 | 1997 | Post-reintroduction | Red kite | Bone collagen | -23.9 | 1.37 | -22.5 | 7.9 | 50.5 | 17.6 | 3.3 |
| RK40 | DB16.10 | 2006 | Post-reintroduction | Red kite | Bone collagen | -23.8 | 1.63 | -22.1 | 9.7 | 51.4 | 17.2 | 3.5 |
| RK41 | GF93197 | 1999 | Post-reintroduction | Red kite | Bone collagen | -23.3 | 1.45 | -21.9 | 8.6 | 49.6 | 17.3 | 3.4 |
| RK42 | GF19084 | 2007 | Post-reintroduction | Red kite | Bone collagen | -23.2 | 1.65 | -21.5 | 9.7 | 50.3 | 17.7 | 3.3 |
| RK44 | GN57326 | 2003 | Post-reintroduction | Red kite | Bone collagen | -23.1 | 1.54 | -21.5 | 8.8 | 52.0 | 17.1 | 3.6 |
| RK45 | NMS.Z.1994.85 | 1993 | Post-reintroduction | Red kite | Bone collagen | -21.8 | 1.24 | -20.6 | 10.5 | 50.7 | 18.1 | 3.3 |
| RK46 | GJ78743 | 2005 | Post-reintroduction | Red kite | Bone collagen | -22.6 | 1.6 | -21.0 | 9.3 | 50.9 | 17.6 | 3.4 |
| RK47 | GN48506 | 2001 | Post-reintroduction | Red kite | Bone collagen | -23.6 | 1.46 | -22.1 | 8.8 | 53.2 | 17.4 | 3.6 |
| RK48 | HT89260 | 1998 | Post-reintroduction | Red kite | Bone collagen | -23.1 | 1.42 | -21.7 | 10.4 | 52.6 | 17.5 | 3.5 |
| RK49 | NMS.Z.1995.86 | 1994 | Post-reintroduction | Red kite | Bone collagen | -22.3 | 1.27 | -21.0 | 10.0 | 50.1 | 17.8 | 3.3 |
| RK50 | NMS.Z.1994.9.3 | 1992 | Post-reintroduction | Red kite | Bone collagen | -23.6 | 1.24 | -22.3 | 8.9 | 53.3 | 17.5 | 3.6 |
| RK51 | DB74.00 | 1999 | Post-reintroduction | Red kite | Bone collagen | -23.4 | 1.45 | -22.0 | 10.0 | 49.0 | 17.2 | 3.3 |
| RK52 | GN22144 | 2003 | Post-reintroduction | Red kite | Bone collagen | -23.3 | 1.54 | -21.8 | 9.9 | 51.6 | 17.1 | 3.5 |
| RK53 | DB73.00 | 1999 | Post-reintroduction | Red kite | Bone collagen | -23.4 | 1.45 | -21.9 | 9.2 | 49.1 | 17.1 | 3.4 |
| RK54 | NS760955 | 2001 | Post-reintroduction | Red kite | Bone collagen | -23.6 | 1.46 | -22.2 | 9.3 | 52.0 | 17.0 | 3.6 |
| RK55 | 351/97 | 1997 | Post-reintroduction | Red kite | Bone collagen | -22.7 | 1.37 | -21.4 | 9.1 | 49.5 | 17.3 | 3.4 |
| RK56 | GF28870 | 1995 | Post-reintroduction | Red kite | Bone collagen | -22.9 | 1.31 | -21.6 | 10.3 | 51.2 | 17.2 | 3.5 |
| WTE1 | NMS.Z.2011.65.2 | 2003 | Post-reintroduction | White-tailed eagle | Bone collagen | -20.6 | 1.54 | -19.0 | 11.1 | 42.3 | 14.5 | 3.4 |
| WTE2 | NMS.Z.1999.112.2 | 1993 | Post-reintroduction | White-tailed eagle | Bone collagen | -20.3 | 1.24 | -19.1 | 9.1 | 40.8 | 14.5 | 3.3 |
| WTE3 | CAT203 | 1894 | Pre-reintroduction | White-tailed eagle | Bone collagen | -15.5 | 0.11 | -15.4 | 13.5 | 41.2 | 14.9 | 3.2 |
| WTE4 | B55194 | 1994 | Post-reintroduction | White-tailed eagle | Bone collagen | -21.9 | 1.27 | -20.6 | 11.0 | 41.0 | 14.3 | 3.3 |
| WTE5 | NMS.Z.2010.87 | 2010 | Post-reintroduction | White-tailed eagle | Bone collagen | -22.7 | 1.7 | -21.0 | 9.1 | 42.4 | 15.3 | 3.2 |
| WTE7 | NMS.Z.2011.92 | 2000 | Post-reintroduction | White-tailed eagle | Bone collagen | -21.5 | 1.45 | -20.0 | 9.2 | 38.9 | 13.7 | 3.3 |
| WTE8 | B102218 | 2013 | Post-reintroduction | White-tailed eagle | Bone collagen | -21.9 | 1.78 | -20.1 | 8.6 | 40.4 | 14.4 | 3.3 |
| WTE16 | NMS.Z.2011.119 | 2009 | Post-reintroduction | White-tailed eagle | Bone collagen | -20.0 | 1.68 | -18.3 | 12.5 | 41.3 | 14.9 | 3.2 |
| WTE17 | NMS.Z.2000.23 | 1998 | Post-reintroduction | White-tailed eagle | Bone collagen | -21.2 | 1.42 | -19.8 | 9.7 | 39.6 | 14.1 | 3.3 |
| WTE18 | B601864 | *2010* | Post-reintroduction | White-tailed eagle | Bone collagen | -21.5 | 1.7 | -19.8 | 11.7 | 42.6 | 15.0 | 3.3 |
| WTE19 | CAT718 | *1900* | Pre-reintroduction | White-tailed eagle | Bone collagen | -20.2 | 0.12 | -20.0 | 10.4 | 42.8 | 15.6 | 3.2 |
| WTE20 | GH257.13 | 2011 | Post-reintroduction | White-tailed eagle | Bone collagen | -20.3 | 1.72 | -18.5 | 11.1 | 41.2 | 14.9 | 3.2 |
| WTE21 | SR37.16 | *2015* | Post-reintroduction | White-tailed eagle | Bone collagen | -18.7 | 1.83 | -16.9 | 12.7 | 42.1 | 14.7 | 3.3 |
| WTE22 | SR4.17 | *2015* | Post-reintroduction | White-tailed eagle | Bone collagen | -20.3 | 1.83 | -18.5 | 11.5 | 42.9 | 14.5 | 3.4 |
| WTE23 | "No.6" | *2015* | Post-reintroduction | White-tailed eagle | Bone collagen | -19.6 | 1.83 | -17.7 | 12.6 | 38.2 | 12.5 | 3.6 |
| WTE24 | ZZ1386 | 2011 | Post-reintroduction | White-tailed eagle | Bone collagen | -20.3 | 1.72 | -18.5 | 11.4 | 40.6 | 14.7 | 3.2 |
| WTE25 | NMS.Z.2010.60 | 2008 | Post-reintroduction | White-tailed eagle | Bone collagen | -20.1 | 1.67 | -18.4 | 13.6 | 43.6 | 14.5 | 3.5 |
| WTE26 | NMS.Z.2008.117 | 2008 | Post-reintroduction | White-tailed eagle | Bone collagen | -17.6 | 1.67 | -15.9 | 14.2 | 39.2 | 14.2 | 3.2 |
| WTE27 | NMS.Z.2013.32.2 | 2012 | Post-reintroduction | White-tailed eagle | Bone collagen | -21.2 | 1.75 | -19.5 | 10.3 | 40.2 | 14.2 | 3.3 |
| WTE28 | ZZ1972 | *2011* | Post-reintroduction | White-tailed eagle | Bone collagen | -19.6 | 1.72 | -17.9 | 11.7 | 42.9 | 14.5 | 3.4 |
