# Supplementary material for: The return of raptors to Scotland’s skies: Investigating the diets of reintroduced red kites and white-tailed eagles using stable isotopes
Source: PLoS One. 2025 Jan 8;20(1):e0315945. doi: 10.1371/journal.pone.0315945 (PMC11709231; doi:10.1371/journal.pone.0315945)
Supplement: S2 Table — Abbreviations are: CR = range in δ13C values; NR: = range in δ15N values; TA = total area of the convex hull area that encompasses all data points (‰2); CD = mean distance to centroid; NND = mean nearest neighbour distance; SDNND = standard deviation of nearest neighbour distance. All birds were sampled post-reintroduction. (DOCX) [file pone.0315945.s002.docx]

**Table S2. Layman’s metrics calculated for stable isotope values (‰) of carbon (δ^13^C) and nitrogen (δ^15^N) of bone collagen and feather keratin of red kites *Milvus milvus* and white-tailed eagles *Halieaeetus albicilla*.** Abbreviations are: CR = range in δ^13^C values; NR: = range in δ^15^N values; TA = total area of the convex hull area that encompasses all data points (‰^2^); CD = mean distance to centroid; NND = mean nearest neighbour distance; SDNND = standard deviation of nearest neighbour distance. All birds were sampled post-reintroduction.

| **Species** | **Tissue** | ***n*** | **CR** | **NR** | **TA** | **CD** | **MNND** | **SDNND** |
| --- | --- | --- | --- | --- | --- | --- | --- | --- |
| Red kite | Bone collagen | 36 | 2.3 | 3.6 | 5.7 | 0.9 | 0.3 | 0.2 |
|  | Feather keratin | 27 | 3.3 | 4.5 | 9.1 | 1.2 | 0.4 | 0.3 |
| White-tailed eagle | Bone collagen | 18 | 5.1 | 5.6 | 11.7 | 1.7 | 0.7 | 0.4 |
|  | Feather keratin | 7 | 3.5 | 4.5 | 5.2 | 2.1 | 0.7 | 0.4 |
